# Biological Hallmarks and New Therapeutic Approaches for the Treatment of PDAC

**DOI:** 10.3390/life11080843

**Published:** 2021-08-18

**Authors:** Graziana Digiacomo, Francesco Volta, Ingrid Garajova, Rita Balsano, Andrea Cavazzoni

**Affiliations:** 1 Department of Medicine and Surgery, University of Parma, 43126 Parma, Italy; francesco.volta@unipr.it (F.V.); andrea.cavazzoni@unipr.it (A.C.); 2 Medical Oncology Unit, University Hospital of Parma, 43100 Parma, Italy; igarajova@ao.pr.it (I.G.); rita.balsano@studenti.unipr.it (R.B.)

**Keywords:** pancreatic ductal adenocarcinoma (PDAC), CDK4/6, KRAS, PD-L1, chemotherapy

## Abstract

Pancreatic Ductal Adenocarcinoma (PDAC) is one of the deadliest solid tumors and is estimated to become a leading cause of cancer-related death in coming years. Despite advances in surgical approaches and the emergence of new chemotherapy options, its poor prognosis has not improved in the last decades. The current treatment for PDAC is the combination of cytotoxic chemotherapy agents. However, PDAC shows resistance to many antineoplastic therapies with rapid progression. Although PDAC represents a heterogeneous disease, there are common alterations including oncogenic mutations of KRAS, and the frequent inactivation of different cell cycle regulators including the CDKN2A tumor suppressor gene. An emerging field of investigation focuses on inhibiting the function of proteins that suppress the immune checkpoint PD-1/PD-L1, with activation of the endogenous immune response. To date, all conventional immunotherapies have been less successful in patients with PDAC compared to other tumors. The need for new targets, associated with an extended molecular analysis of tumor samples could give new pharmacological options for the treatment of PDAC. It is, therefore, important to push for a broader molecular approach in PDAC research. Here, we provide a selected summary of emerging strategy options for targeting PDAC using CDK4/6 inhibitors, RAS inhibitors, and new drug combinations with immune checkpoint agents.

## 1. Introduction

Pancreatic ductal adenocarcinoma (PDAC) is associated with a very poor prognosis and the overall five-year survival rate is about 6% [1], with growing mortality rates in the next decade [2]. Radical surgical resection remains the only potentially curative option for PDAC patients; however, the recurrence rate is as high as 85% [3]. Interestingly, an autopsy series showed that 30% of patients died with locally destructive PDAC without the widespread metastatic disease [4,5]. Unfortunately, progresses in the management of both locally advanced and metastatic PDAC have been very modest in the last decades. Currently, polychemotherapy regimens (gemcitabine and abraxane or FOLFIRINOX) are the gold standard of treatment in this setting, though providing only slight improvements in PDAC patients’ outcomes [6,7]. The median overall survival (OS) of metastatic or locally advanced PDAC patients treated with the FOLFIRINOX regimen is 11.1 months, with median progression-free survival (PFS) of 6.4 months and the percentage of objective response rate is 31.6 [6]. For PDAC patients treated with gemcitabine and abraxane, the median OS reaches 8.5 months and the median PFS is 5.5 months. The response rate remains less than in the third of all patients (23%) [7]. The implementation of genetic testing might change a very narrow treatment landscape for small subsets of PDAC patients: in particular, in BRCA1/2 mutated settings, olaparib (PARP inhibitor) might be proposed as a maintenance strategy [8]. No target therapies and no immunotherapy approaches are nowadays clearly effective in PDAC. For this reason, novel therapeutic options and multi-target approaches are urgent in order to fight PDAC cancer.

In this review, we provide a comprehensive summary of three different therapeutical approaches for PDAC, in particular, the development of new strategies to target KRAS, the use of specific CDK4/6 inhibitors, and finally, the combination of immune checkpoint inhibitors with chemotherapy or molecular targeted agents.

## 2. KRAS Pathway

**Figure 1:**
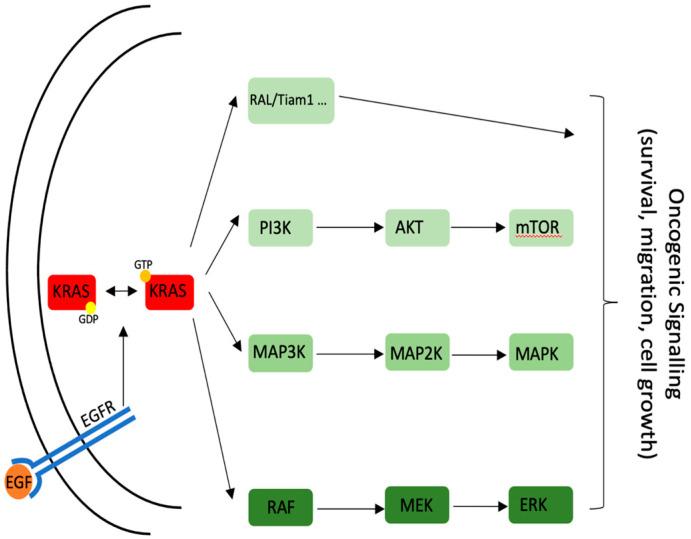
KRAS-related signaling and downstream effector proteins.

KRAS encodes for a highly conserved protein with GTPase activity [9] and switches between an active GTP-bound and an inactive GDP-bound state; the switch is mediated by different guanosine exchange factors (GEFs). The different pathways that can originate from KRAS are illustrated in Figure 1. All the mutations found in PDAC are activating ones and block the inactivation of KRAS [10,11] maintaining KRAS in a constitutive, GTP-bound state.

One of the upstream effectors of KRAS is the Epidermal Growth Factor Receptor (EGFR). In contrast with other tumors such as lung cancer, in PDAC, activating mutations in EGFR have never been found. However, a higher expression of this receptor has been linked with shorter patient survival [12].

Interestingly, when a second mutation in TP53 is present, activation of EGFR is not necessary for RAS induction [13]. For this reason, targeted therapy for the treatment of EGFR (an obvious choice due to the high number of compounds available for other neoplastic syndromes and the fact that it is upstream of KRAS) using Erlotinib has never brought positive results [14].

Of note, in lung cancer, the mutations in EGFR and KRAS (the two most common ones) are considered mutually exclusive [15]. It is, therefore, possible that the high incidence of KRAS mutations in PDAC is the reason why EGFR has never been found mutated in this kind of tumor. Several pathways originate downstream of KRAS. Some of the most important downstream KRAS proteins are shown in Figure 1 [16]. However, it is not the purpose of this review to explain in detail every single player downstream of KRAS. For clinical reasons, it is important to underline the high level of crosstalk between the different pathways.

Several efforts in targeting one or more of the KRAS downstream effectors failed, due to the high redundancy level of this signaling.

### 2.1. Mutational Status

KRAS is considered mutated in more than 90% of PDAC. The glycine in position 12 is the most frequently modified site. G12D accounts for 41% of all KRAS mutations, G12V for 24%, and G12R for 16%; overall, they account for more than 80% of cases. Another notable mutation is the Q61X that accounts for another 10%. All the previously mentioned mutations lead to abnormal activation of KRAS that is unable to de-activate, with a persistent GTP-bound state. Therefore, the downstream signaling cascade and its effects (higher proliferation and invasiveness) are constitutively activated [10,17,18,19,20].

Unfortunately, PDAC is often diagnosed at a late stage; it is therefore common to find secondary mutations that contribute to the onset of the neoplastic mass [21]. The most common genes that are found mutated together with KRAS are TP53 (40–74%), SMAD4 (1–50%), and CDKN2A (5–50%) [10,17,18,19,20,22]; all three genes encode for proteins that regulate cell cycle progression and are commonly found misregulated in different kinds of tumors; extensive research has already been done on their respective pathways.

### 2.2. KRAS Therapy

For all the reasons mentioned above (secondary mutation, redundancy of downstream effectors), KRAS has always been thought to be an “undruggable” candidate.

Recently, several companies focused on finding new compounds that directly target KRAS. These efforts have produced the first inhibitor of G12D mutation with promising results both in the in vivo and in vitro settings [23]. Of note, in the last couple of years, different compounds have been proposed as inhibitors of KRAS G12C mutation in colorectal and lung cancers [24,25]. Even if this discovery will likely not be beneficial to PDAC patients due to the different type of mutations, advancements in targeted therapy are, in any case, reason for optimism in oncology research. 

Recently, McCarthy et al. [26] proposed a new compound with promising KRAS inhibitory ability. This new small molecule, pyrazolopyrimidine-based, binds both wild-type and mutant KRAS. It has the advantage of a high degree of inhibitory capacity disrupting the downstream signaling such as MAPK and Raf, and it is not directed against one specific mutation but has a broad affinity. Moreover, in contrast to other inhibitors, this compound blocks also the Akt pathway, which is usually upregulated upon KRAS inhibition [27]. The data above-mentioned were collected in preclinical models. They showed promising results that need to be translated in clinical trials. In particular, it is necessary to assess the effect on the non-cancer cells that carry a wild-type form of KRAS that will be inhibited as well. 

Due to the difficulties of directly targeting RAS (proved by the lack of inhibitors for other types of mutations), different strategies have been tested. Since RAS proteins, to act properly, need to be localized close to the plasma membrane and this localization is mediated by a modification performed by an enzyme called farnesyltransferase [28,29], extensive research has been performed trying to block this activity. Tipifarnib was proposed as a farnesyltransferase inhibitor that blocks the bind of RAS protein to the membrane, inhibiting the prenylation of the CAAX motif. Unfortunately, despite preclinical promising results, the clinical outcome was not impressive [30].

In the last decades, several pathways have been targeted, thanks to the availability of small, new molecules that can inhibit certain parts of the protein that were not targetable with traditional drugs. Thanks to this new strategy, it is now possible to try to block the exchange between GDP and GTP in the pocket of RAS protein [31]. SCH53239 and SCH54292 compounds act exactly in this way, by maintaining KRAS in the GDP-bound inactive state, with the arrest of the proliferation of tumor cells [32]. Unfortunately, the high level of toxicity does not make them a good strategy of intervention in clinics.

More recently [33], by in silico screen, new small molecules, Kobe0065 and Kobe2602, that act on the binding between HRAS in the GTP bound form and Raf1 were identified. Their antitumor activity was shown in colon cancers but not in pancreatic ones. However, all the downstream pathways of RAS (AKT, RAF, MEK) were downregulated, making these small molecules promising compounds for further studies. 

Targeting the GTP/GDP pocket of KRAS was also the purpose of AGP (Androprapholide). Preclinical promising results (apoptosis in pancreatic cancer cells and synergistic effect with gemcitabine [34]) were not followed by positive clinical results. Only one clinical study was presented in colorectal cancer but without any efficacy reported [35].

### 2.3. Clinical Trials

The ongoing clinical trials targeting KRAS in PDAC reflect the lack of suitable inhibitors (Table 1). The only KRAS-directed trial uses a G12C specific inhibitor developed by Mirati Therapeutics [25]. It is, however, important to remember that G12C is a mutation found with low frequency in PDAC. Other direct KRAS inhibitors are not present in clinical trials. However, other ways to target this protein could be of interest. For example, a study from the MD Anderson in Houston contemplates the use of exosomes carrying a siRNA against G12D mutated KRAS. The technology, per se, could also be adaptable to different kinds of mutations and tumors or another branch of the pathway, as proposed by Nitto Biopharma in a study using siRNA against GSTP (glutathione s-Transferase P), a modulator of RAS proteins.

## 3. CDK4/6 Pathway

KRAS is considered the main initiator of oncogenesis in pancreas. After a first mutation in KRAS, secondary ones in cell cycle regulators can contribute to the development of the tumor and the progression to a more aggressive one [22]. One of the most frequent mutations reported in PDAC patients concerns the CDK2A gene. 

The cyclin-dependent kinase inhibitor 2A (CDKN2A) gene is located on chromosome 9p21.3 and encodes for two cell cycle regulatory proteins: cyclin-dependent kinase inhibitor 2A (p16INK4a) and alternate reading frame (p14ARF) [36]. The p16INK4a protein, which belongs to the INK4 family of cyclin-dependent kinase inhibitors, prevents the formation of the complex cyclin D1/CDK4/6 that regulates Rb function. CDK4/cyclin D1, by phosphorylating Rb1 protein, induces the release of the transcription factor E2F, which activates genes involved in the progression from G1 to S phase [37,38,39]. In contrast, Rb in its hypophosphorylated active form leads to G1 cell cycle arrest by sequestering the E2F protein. The alteration of the Rb pathway leads to the pathologic progression of the cell cycle in the S phase and can be caused by the loss of p16INK4a, the amplification of cyclin D1, or the failure to express Rb1 [40]. 

### 3.1. Mutational Status

Mutations of cyclin-dependent kinase inhibitor 2A is frequently found in PDAC patients. Previous studies have reported that CDKN2A is inactivated in about 95% of PDAC by different mechanisms such as homozygous deletion of both alleles, intragenic mutation in one allele or promoter hypermethylation. CDKN2A is inactivated in about 40% of cases by deletion of both alleles [41,42,43,44]; another 15% of PDAC shows hypermethylation of the promoter sequence for CDKN2A [41,45,46,47]. Moreover, further data confirmed the role of this mutation on the PDAC progression to a more aggressive phenotype. Metastatic pancreatic cancer shows a much higher deletion rate of p16INK4a protein compared with nonmetastatic PDAC [48]. The presence of about 40% of germline mutations of CDKN2A causes FAMMM (Familial atypical multiple mole melanoma), an autosomal, dominantly inherited disorder characterized by multiple nevi and atypical nevi, and an increased risk for malignant melanoma [49]; patients with FAMMM syndrome have an increased risk of about 50% of developing pancreatic cancer [50]. 

### 3.2. CDK4/6 Therapy

As the CDK4/6-Cyclin D pathway is frequently deregulated in the majority of PDAC patients by CDKN2A inactivation, the use of specific CDK4/6 inhibitors in PDAC could be a promising novel approach. To date, three CDK4/6 inhibitors, palbociclib, abemaciclib, and ribociclib are approved for patients with Estrogen Receptor (ER)-positive, Human Epidermal Growth Factor Receptor 2 (HER)-negative advanced breast cancer in association with endocrine therapy [51]. The effect of CDK4/6 inhibitors is cytostatic in cell culture and animal models of solid tumors; in contrast, the effect of CDK4/6 inhibitors is cytotoxic in selected hematological diseases [52]. In preclinical models, the inhibition of CDK4/6 induces Rb function and promotes senescent-like arrest [53]. Abemaciclib showed more potency than the two other compounds with higher selectivity for CDK4 and CDK9 [54]. Abemaciclib inhibits Rb phosphorylation, resulting in cell cycle arrest in G1 and inhibition of cell growth only in Rb-proficient cells [55]. Clinical studies have demonstrated that CDK4/6 inhibitors alone present drug activity in some tumor models as observed in liposarcoma [56]. Numerous studies have confirmed that CDK4/6 inhibitors in monotherapy have a limited antitumor effect; for this reason, to improve the cytostatic effect of this class of compounds, recent strategies focused on the combination of CDK4/6 inhibitors with other compounds. Several clinical studies are evaluating the benefit of CDK4/6 inhibition alone or in association with targeted drugs or immune checkpoint inhibitors in PDAC patients (Table 2). In the preclinical setting, a synergism between abemaciclib and yes-associated protein 1 (YAP1) or human antigen R (HuR) inhibitors was observed in PDAC cells [57]: this synergism can be explained by the fact that both drugs act on cyclinD1/CDK4/6 axis [57]. In addition, YAP1 inhibition synergizes with CDK6 targeting agents in esophageal cancer both in vitro and in vivo systems: both inhibition of YAP1 and CDK6 pathways significantly reduced esophageal cancer cell growth and showed a strong antitumor effect in vivo against radiation-resistant esophageal cancer cells [58]. Moreover, dual inhibition with palbociclib (CDK4/6 inhibitor) and trametinib (MEK inhibitor), which acts downstream of KRAS, has demonstrated efficacy in xenograft models of colorectal cancer [59]; this combination modulates the PDAC microenvironment through the increase of the sensitivity of PDAC cells to immune checkpoint blockade [60]. In a preclinical study, the combination between MEK and CDK4/6 inhibitors prevents tumor cell proliferation by promoting senescence in vitro, without any significant change in apoptosis in PDAC cells [61]. The CDK4/6 inhibitors have also been proposed in association with chemotherapy agents; in particular, the addition of CDK4/6 inhibitors to gemcitabine exerts a synergistic effect in PDAC cells [62] with increased apoptosis and chemosensitivity, reduction of both invasion and tumor progression.

One of the mechanisms regulating chemotherapy resistance in PDAC is metabolic deregulation. Previous studies have reported that palbociclib reduces aerobic glycolysis through the stabilization of fructose-1,6-bisphosphatase (FBP1) [63]. Data reported that FBP1 is transcriptionally downregulated by multiple factors including Snail [64] nucleophosmin-1 (NPM1) [65] and hepatocyte nuclear factor 4 alpha (HNF4α) [66]. Moreover, FBP1 is post-transcriptionally modulated by the MAGE-tripartite motif-containing 28 (TRIM28) complex and USP44 in PDAC and liver cancers [67]. Under regular conditions, many Melanoma Antigen Gene (MAGE) proteins are expressed in the reproductive organ. They are often overexpressed in several malignant lesions [68]: as reported [61], MAGED1 was overexpressed in PDAC patients, and the overexpression was associated with poor prognosis. Recently, it has been demonstrated that the TRIM28-MAGE complex degrades FBP1 in liver cancer cells [69]. Therefore, based on the fact that the CDK4/6-E2 F1 axis is required to increase MAGED1 expression with a concomitant degradation of FBP1, the authors suggested that CDK4/6 inhibition could eliminate the aerobic glycolysis effect through the stabilization of FBP1 in PDAC [63].

## 4. Effect of Immunotherapy in Pancreatic Cancer

The recent breakthrough of immune checkpoint inhibition in cancer treatment produced a vast impact on the outcome of patients, in particular for melanoma and Non-Small Cell Lung Cancer (NSCLC) [70,71], but, at present, single agents or combined Immune Checkpoint Inhibitors (ICI) do not offer clinical benefit to PDAC patients. In recent years, the use of monotherapy-based treatment with antibody targeting Cytotoxic T-Lymphocyte Antigen 4 (CTLA-4) and Programmed cell death protein 1(PD-1/PD-L1) signaling has failed. In particular, with the anti-CTLA-4 antibody ipilimumab, no objective response rate was reported in Phase II clinical trials [72]. Similarly, the anti-PD-1 antibody pembrolizumab failed to make any significant results in monotherapy in a Phase I pan-cancer study [73]. Likewise, the anti-PD-L1 agent durvalumab [74], in phase II clinical trials, did not produce significant results in PDAC patients previously treated with fluorouracil–based or gemcitabine-based treatment, as a single regimen or coupled with the anti-CTLA-4 inhibiting antibody tremelimumab, suggesting that the combined regimen with immune targeting agent to both PD-1/PD-L1 and CTLA-4 axis did not produce any significant results in terms of overall survival.

The lack of response to ICI in monotherapy is mainly ascribed to the absence of CD3+ T-cells’ infiltrate in the tumor mass; as reported [75,76], the stroma of PDAC exerts its trapping function to CD3+ T-cells, with physical separation of tumor and immune cells. As a consequence, the tumor stroma in PDAC patients presents an immunosuppressive phenotype. A strategy used to improve the efficacy of ICIs is the combination of ICIs with standard chemotherapy regimens or targeted agents as reported for NSCLC [77,78] or renal cell carcinoma [79,80]. The same strategy has been tested in PDAC to act on the tumor microenvironment and to favor the accumulation of both CD4+ and CD8+ T-cells. As reported [81], some clinical trials have been evaluating the combination of gemcitabine and nab-paclitaxel, in association with anti-PD-L1/PD-1 and/or anti-CTLA-4 agents. Chemotherapy alone may also alter the immune setting of the tumor microenvironment in PDAC. Interestingly, preoperative therapy with chemoradiotherapy or chemotherapy has shown the ability to reduce both myeloid-derived suppressor cells (MDSCs) and Treg infiltrates, the main cause of immune evasion, leading to an increase in the CD8 T-cells: T-reg ratio [82]. Similarly, a different study showed that neoadjuvant chemoradiotherapy can increase T-cell infiltrates, which represent a stronger predictor of outcomes than pathologic response to treatment [83].

Based on these results, some clinical trials evaluating the combination of chemotherapy with ICIs are ongoing (Table 3). Three phase III clinical trials enrolling PDAC patients receiving immune checkpoint inhibitors combined with chemotherapy are in progress. In detail, in the NCT03977272 clinical trial, 110 stage IV patients with pancreatic cancer are enrolling and the standard of care treatment, based on FOLFIRINOX (Folic acid, Irinotecan, Oxaliplatin, and 5-Fluorouracil), will be compared to the combination of FOLFIRINOX with an anti-PD-1 agent. The NCT03983057 trial is recruiting patients with borderline resectable and locally advanced pancreatic cancer to compare the effects of FOLFIRINOX regimen alone or combined with an anti-PD-1 agent, with the progression-free survival as the primary endpoint. Finally, in the NCT04674956 study, the combination of the anti-PD-1 agent camrelizumab with the chemotherapy regimen gemcitabine/nab-paclitaxel, is compared with the dual chemotherapeutic agents and the progression-free survival is the primary endpoint.

Several studies have focused on the combination of ICI with specific targeted agents mainly directed to the tumor microenvironment, as reported in Table 4.

Much of the current research is focused on understanding the immunosuppressive ability of the tumor microenvironment (TME) that leads to immune evasion and how the TME plays its role in limiting the effectiveness of immunotherapy. One of the most critical components of TME is the extracellular matrix (ECM). Whether it is the structural components of the ECM or the immune suppressive cells that promote resistance to ICI, the TME is a primary mediator of tumor development and resistance to drug treatment or ICIs. The TME is composed of a variety of cells such as activated pancreatic stellate cells, endothelial cells, infiltrating immune cells (myeloid-derived suppressor cells, T-reg cells), and tumor-associated macrophages, and finally, extracellular matrix components, mainly produced by stellate cells. This cell population is responsible for desmoplasia, a pathological process associated with the high production of extracellular matrix, in particular, type I collagen. The peculiar organization of TME in PDAC cancers is probably responsible for an immune-suppressive phenotype. The matrix density [84] is able to reduce CD8+ T-cell growth and tumor infiltration. One of the most attractive targets involved in the connection between ECM and intracellular signaling is the focal adhesion kinase (FAK) [85]. Activated FAK mediates many intra- and extracellular processes involved in tumor cell migration and invasion, from cell adhesion to ECM remodeling, in addition to the expression of matrix metalloproteinases [86,87]. Moreover, the high collagenase I level in ECM secreted by cancer-associated fibroblasts (CAF) correlates with high FAK activation and increase of stem cells’ properties, associated with high β1-integrin signaling [88,89]. Targeting FAK kinase, in association with other therapeutic options in pancreatic tumors could be an attractive strategy. As reported [90], FAK inhibition synergizes with nab-paclitaxel in preclinical models of PDAC cells. Moreover [91,92], in preclinical PDAC models, the inhibition of FAK kinase sensitizes cancer cells to radiotherapy, enhancing CD8+ T-cell infiltration in the TME and a consequent reduction of granulocyte population. In addition to previous data, FAK inhibition improves the effect of ICI compounds [93,94,95]. In particular, Jiang and collaborators demonstrated that FAK inhibition in an in vivo model of PDAC cancer [93] produced a significant shift from immune suppressive cells to CD8+ T-cells in the tumor infiltrate. FAK inhibition in cancer cells can influence the composition of TME, with a shift from a “cold” to a “hot” TME. This is a relevant aspect for ICI success. In fact, in the same manuscript, FAK inhibition improves the effect of ICI on in vivo murine model of PDAC. As the immune escape of pancreatic cancer cells is mainly attributed to its special tumor environment [96], targeting the stroma of tumors represents a new strategy to fight PDAC. FAK inhibition is one of the aforementioned strategies, but emerging therapies are directed to specific TME components. Another signaling involved in pancreatic cancer progression is the transforming growth factor-beta (TGF-β) pathway [97]. TGF-β is reported to play a context-dependent, yet contradictory role: on one hand, it presents tumor-suppressive properties in early, non-metastatic PDAC and it is responsible for tumor progression in advanced and metastatic PDAC [98]. TGF-β exerts its tumor suppressor properties in a SMAD4 dependent manner [99]. On the other hand, in advanced PDAC tumors, TGF-β is reported to promote tumor progression, by a non-SMAD4 dependent signaling, via the activation of the WNT/β-catenin axis, known to regulate numerous biological and pathological processes [100]. Currently, three clinical trials included the TGF-β signaling, as a target for advanced PDAC are ongoing: the phase I NCT00844064, where the safety and tolerability of the antisense oligo-deoxynucleotide AP 12009 (trabedersen) directed toward TFG-β2 mRNA is evaluated. In the phase I/II clinical trial NCT03451773, the dual TFG-β/PD-L1 binding molecule M7824 is evaluated in combination with gemcitabine in advanced pancreatic cancer. Finally, as reported in Table 4, in the phase I NCT02734160 trial, the TGFRI inhibitor galunisertib is evaluated in combination with the ICI pembrolizumab. Interestingly, in two of three trials, the rationale is to target TGF-β, and the immune checkpoint PD-1/PD-L1, as a promising strategy for PDAC patients. Parallel inhibition of both pathways is a viable strategy to increase T-cell infiltration and cytotoxicity, as reported by the employ of nanoparticles carrying the TGF-β inhibitor LY2157299 and a siRNA specific for PDL1 [101]. 

Finally, recent insights on the role of KRAS in PDAC [102] demonstrated that KRAS knock-out cells increase the input of immune cells into the TME, with higher expression of immune checkpoint components that may cause suppression of T-cell response. These data imply that the anticancer immune response is critical for therapeutic management of KRAS-driven tumors and that the combination regimen toward KRAS signaling and the immune checkpoint could be a promising strategy to treat PDAC.

## 5. Discussion

This review provides a brief overview of preclinical and clinical studies focusing on novel therapeutic options concerning specific KRAS, CDK4/6 inhibitors, and combination of immune checkpoint inhibitors with chemo or molecular targeted agents for the treatment of pancreatic cancer.

Emerging strategies to fight PDAC, such as the use of immunotherapeutic vaccines have already been discussed in recent reviews [79,103,104].

As reported in many clinical reports, the current interventional therapy is based on the combination of chemotherapeutic agents, as FOLFIRINOX or gemcitabine-based strategies, providing only slight improvements in PDAC patients’ outcomes [6,7]. The effect of targeted therapy and the use of the ICI in advanced pancreatic cancer failed. For this reason, it is urgent to develop novel strategies.

Since PDAC presents a complex mutational status, it is important to use a wide approach in developing new drugs. A single-drug approach will likely fail in improving patient outcomes.

The purpose of this review is, therefore, to summarize the novel therapeutic options on three different intervention strategies and encourage a multi-target approach.

With the discovery of specific agents targeting oncogenic forms of KRAS [23,24], new therapeutic lines of intervention could be promoted with the use of these new compounds, in monotherapy or eventually in combination with other drugs. Moreover, several clinical trials involving the use of CDK4/6 inhibitors alone, or in combination with chemotherapy or targeted agents could represent a new frontier for the targeting of PDAC patients. 

In the last part of this review, we explore the role of ICIs, and clinical trials of phases II and III are reported, where immune checkpoints’ inhibitors are combined with other agents, such as FOLFIRINOX or gemcitabine (Table 3) or specific targeted agents (Table 4).

The use of ICI alone in PDAC has been challenging, but new therapeutic options and combination strategies are beginning to show promising results in preclinical settings and finally, in clinical trials. 

Since these results are mostly still at the exploratory level, several obstacles remain to be overcome. For the success of future trials and preclinical data, an improved understanding of the dynamic tumor microenvironment is necessary. In this way, it will be possible to identify new strategies to overcome the resistance of PDAC to single-agent immune checkpoint treatment. 

## Figures and Tables

**Table 1:** Clinical trials with KRAS targeting inhibitors (http://clinicaltrials.gov/, accessed on 1 June 2021).

Agent	Target	Combination	Phase	Reference
MRTX849	KRAS G12C	-	I/II	NCT03785249
Exosome G12D siRNA	KRAS G12D	-	I	NCT03608631
NBF-006	KRAS	-	I	NCT03819387

**Table 2:** Clinical trials with CDK4/6 targeting agents coupled with other targeted drugs or PD-1/PD-L1 inhibitors (http://clinicaltrials.gov/, accessed on 1 June 2021).

Agent	Target	Combination	Phase	References
Palbociclib	CDK4/6	Ulixertinib	I	NCT03454035
Palbociclib	CDK4/6	Gedatolisib	I	NCT03065062
Palbociclib	CDK4/6	Carboplatin Cisplatin	I	NCT02897375
Palbociclib	CDK4/6		II	NCT02806648
Palbociclib	CDK4/6		II	NCT02465060
Abemaciclib Palbociclib	CDK4/6		I	NCT03878524
Abemaciclib	CDK4/6	LY3023414 Gemcitabine Capecitabine	II	NCT02981342
Abemaciclib	CDK4/6		II	NCT03891784
Abemaciclib	CDK4/6	LY3300054	I	NCT02791334
Ribociclib	CDK4/6	Trametinib	I/II	NCT02703571
Ribociclib	CDK4/6		II	NCT02420691

**Table 3:** Phase II/III clinical trials with standard chemotherapy agents coupled with PD-1/PD-L1 inhibitors (http://clinicaltrials.gov/, accessed on 1 June 2021).

Agent	Combination	Phase	Reference
FOLFIRINOX	Anti PD-1	III	NCT03977272
Gemcitabine or FOLFIRINOX	Pembrolizumab	II	NCT04447092
Gemcitabine or S-1	Nivolumab	II	NCT04377048
FOLFIRINOX	BsAb PD-1/CTLA-4	I/II	NCT04324307
Gemcitabine/nab-paclitaxel	Nivolumab/Ipilimumab	I/II	NCT04247165
FOLFIRINOX	Nivolumab	I/II	NCT03970252
FOLFIRINOX	Anti PD-1	III	NCT03983057
Gemcitabine/nab-paclitaxel	Camrelizumab	III	NCT04674956

**Table 4:** Clinical trials with molecular targeting agents coupled with PD-1/PD-L1 inhibitors (http://clinicaltrials.gov/, accessed on 1 June 2021).

Agent	Target	Combination	Phase	Reference
Anlotinib (AK105)	Multi-kinase	Anti-PD-1	I/II	NCT04803851
Defactinib	FAK	Pembrolizumab	I/II	NCT02758587
LYT-200	Galectin-9	Anti PD-1	I/II	NCT04666688
Anetumab Ravtansine	Mesothelin	Nivolumab Nivolumab/Ipilimumab Nivolumab/gemcitabine	I/II	NCT03816358
Anlotinib	Multi-kinase	Toripalimab	I/II	NCT04718701
Olaptesed pegol (NOX-A12)	CXCL12	Pembrolizumab	I/II	NCT03168139
Entinostat	HDAC	Nivolumab	II	NCT03250273
Galunisertib	TGFβRI	Durvalumab	I	NCT02734160
Merestinib	MET	LY3300054	I	NCT02791334
Pexidartinib	CSF1R	Durvalumab	I	NCT02777710
Danvatirsen	STAT3	Durvalumab	II	NCT02983578
Plerixafor	Hematopoietic stem cells	Cemiplimab	II	NCT04177810
KY1044	ICOS	Atezolizumab	I/II	NCT03829501
Ibrutinib	BTK	Durvalumab	I/II	NCT02403271
Defactinib	FAK	Pembrolizumab	II	NCT03727880
Itacitinib	JAK1	Pembrolizumab	I	NCT02646748

## Data Availability

Not applicable.
